# Hybrid Spike-Encoded Spiking Neural Networks for Real-Time EEG Seizure Detection: A Comparative Benchmark

**DOI:** 10.3390/biomimetics11010075

**Published:** 2026-01-16

**Authors:** Ali Mehrabi, Neethu Sreenivasan, Upul Gunawardana, Gaetano Gargiulo

**Affiliations:** School of Engineering, Western Sydney University, Penrith, NSW 2751, Australia; n.sreenivasan@westernsydney.edu.au (N.S.);

**Keywords:** epileptic seizure, epilepsy, electroencephalography (EEG), real-time signal processing, spiking neural networks (SNNs), convolutional spiking neural networks (Conv-SNNs), biomimetic design

## Abstract

Reliable and low-latency seizure detection from electroencephalography (EEG) is critical for continuous clinical monitoring and emerging wearable health technologies. Spiking neural networks (SNNs) provide an event-driven computational paradigm that is well suited to real-time signal processing, yet achieving competitive seizure detection performance with constrained model complexity remains challenging. This work introduces a hybrid spike encoding scheme that combines Delta–Sigma (change-based) and stochastic rate representations, together with two spiking architectures designed for real-time EEG analysis: a compact feed-forward HybridSNN and a convolution-enhanced ConvSNN incorporating depthwise-separable convolutions and temporal self-attention. The architectures are intentionally designed to operate on short EEG segments and to balance detection performance with computational practicality for continuous inference. Experiments on the CHB–MIT dataset show that the HybridSNN attains 91.8% accuracy with an F1-score of 0.834 for seizure detection, while the ConvSNN further improves detection performance to 94.7% accuracy and an F1-score of 0.893. Event-level evaluation on continuous EEG recordings yields false-alarm rates of 0.82 and 0.62 per day for the HybridSNN and ConvSNN, respectively. Both models exhibit inference latencies of approximately 1.2 ms per 0.5 s window on standard CPU hardware, supporting continuous real-time operation. These results demonstrate that hybrid spike encoding enables spiking architectures with controlled complexity to achieve seizure detection performance comparable to larger deep learning models reported in the literature, while maintaining low latency and suitability for real-time clinical and wearable EEG monitoring.

## 1. Introduction

Epilepsy is a chronic neurological disorder affecting approximately 65–70 million individuals worldwide and is characterized by recurrent, unprovoked seizures resulting from abnormal, synchronous neuronal discharges in the brain [1,2]. Electroencephalography (EEG) remains the gold standard for seizure detection and diagnosis due to its millisecond temporal resolution and ability to capture cortical electrical activity associated with seizure onset and propagation. Characteristic EEG patterns such as spikes, sharp waves, and spike-and-wave complexes are clinically used to identify ictal events and assess their spatial and temporal dynamics. Accurate and timely seizure detection is essential for both clinical management and the development of closed-loop therapeutic systems, including responsive neurostimulation and wearable seizure-alert devices.

However, automated seizure detection in continuous EEG recordings remains a challenging task. EEG signals are inherently nonstationary and often contaminated with muscle, ocular, and environmental artifacts, while seizure events are rare—typically comprising less than 1% of the total recording time. Furthermore, there is significant variability in seizure morphology across patients, and even within a single patient across different episodes [3]. These factors hinder the generalization of data-driven algorithms and contribute to class imbalance problems. In practical deployment scenarios such as bedside monitoring, ambulatory EEG, and implantable systems, additional constraints on latency, power consumption, and real-time responsiveness further complicate model design. Therefore, there is a critical need for detection frameworks that combine high accuracy with computational efficiency and low energy cost.

Traditional EEG-based seizure detection methods rely on handcrafted signal processing and machine learning pipelines. These approaches extract features such as spectral power, entropy, or wavelet coefficients before classification using support vector machines (SVMs), random forests (RFs), or multilayer perceptrons [4,5,6]. While interpretable and efficient, such systems are sensitive to noise, dataset bias, and feature selection. More recently, deep learning models—especially convolutional and recurrent neural networks—have achieved strong performance by automatically learning temporal or spectral representations from raw EEG data [7,8,9]. Yet, their continuous-valued activations and dense computations impose high power requirements, making them less suitable for 24/7 portable monitoring or implantable hardware.

Spiking neural networks (SNNs) represent an emerging neuromorphic alternative that more closely mimics biological neural computation. SNNs communicate via discrete spikes and employ leaky integrate-and-fire (LIF) neurons to integrate inputs over time, naturally capturing temporal dependencies and supporting event-driven computation [10,11]. Because computation occurs only when spikes are present, these models can significantly save energy when deployed on dedicated neuromorphic hardware such as Intel Loihi or SpiNNaker [12,13]. However, effective deployment of SNNs for seizure detection requires careful attention to two central design issues: (i) how to convert continuous EEG signals into informative, sparse spike trains that preserve physiologically meaningful information, and (ii) how to construct spiking architectures that can learn both rapid transient and sustained rhythmic seizure features without excessive computational cost.

This work addresses these challenges by introducing a real-time, neuromorphic-inspired framework for EEG-based seizure detection that emphasizes causal processing, compact model design, and spike-based signal representation.

First, we propose a dual spike encoding scheme that combines Delta–Sigma (Δ–Σ) modulation and stochastic rate coding. Delta–Sigma encoding emphasizes rapid temporal changes in the EEG, such as sharp transients and spike-like events, while rate coding preserves slower amplitude-related dynamics over short temporal intervals. The concatenation of these complementary representations yields a sparse yet information-rich spike stream that is well suited to spiking neural computation under real-time constraints.

Second, we investigate two complementary spiking architectures designed with different complexity–performance trade-offs in mind: (i) a compact Hybrid SNN, which performs temporal integration of the dual-coded spike streams using leaky integrate-and-fire (LIF) neurons, and (ii) a more expressive Convolutional SNN (ConvSNN), which incorporates residual depthwise-separable one-dimensional convolutions and temporal multi-head self-attention (MHSA) prior to a spiking classification head [14,15]. Both models are trained using surrogate-gradient backpropagation with mild regularization to promote stable convergence.

The complete system operates on short, fixed-length EEG segments of 0.5 s (128 samples) with 50% overlap. This design enables fully causal, low-latency inference without reliance on future context, making it suitable for continuous, streaming EEG analysis. Optional temporal smoothing and persistence-based thresholding can be applied at the decision level to reduce transient false positives without violating real-time constraints.

The main contributions of this study are summarized as follows:

(i) A hybrid spike encoding strategy that combines Delta–Sigma and rate coding to generate sparse, event-driven EEG representations.
(ii) Two neuromorphic spiking architectures for seizure detection: a lightweight Hybrid SNN and a higher-capacity ConvSNN integrating convolutional and attention-based temporal modeling.
(iii) A fully causal, real-time EEG processing pipeline based on short overlapping windows, together with standardized preprocessing and labeling.
(iv) A matched comparative evaluation against a 1D-CNN baseline using the same patient-wise data partitions, highlighting trade-offs between detection performance, computational complexity, and suitability for real-time deployment.

This study focuses on the design and evaluation of spike encoding strategies and spiking neural architectures for real-time EEG inference, rather than on population-level seizure classification or prospective clinical validation. All evaluations are conducted under within-patient, streaming conditions using patient-wise data separation. Accordingly, the analysis emphasizes window-level detection fidelity, event-level false-alarm behavior, latency, and computational characteristics relevant to online operation. Cross-patient generalization, long-term prospective validation, and hardware-specific energy measurements are acknowledged as important directions for future work, but are outside the scope of the present study.

The remainder of this paper is organized as follows. Section 2 reviews related work on EEG-based seizure detection, highlighting the evolution from traditional signal processing and machine learning methods to deep learning and neuromorphic approaches. Section 3 describes the proposed framework, including dataset preparation, hybrid spike encoding, and the design of the neural architectures. Section 4 presents the experimental setup, quantitative performance evaluation, and comparative analyses. Section 5 interprets the findings, emphasizing their significance for real-time, low-latency, and energy-efficient clinical deployment. Finally, Section 6 concludes the paper and outlines directions for future research.

## 2. Background

Automated EEG seizure detection has evolved along three broad methodological lines: (i) classical signal processing with engineered features and conventional classifiers, (ii) data-driven machine learning and deep learning, and (iii) neuromorphic/spiking approaches. Each line brings different assumptions about EEG structure, distinct computational trade-offs, and varying suitability for real-time, ambulatory deployment. This section summarizes core techniques, representative results, and practical limitations that motivate the comparative study presented in this paper.

### 2.1. Signal Processing Methods

Conventional pipelines begin with preprocessing (e.g., band-pass filtering, notch filtering for 50/60 Hz, artifact attenuation via ICA) followed by hand-crafted feature extraction. Time-domain features (variance, line length, energy, zero-crossing rate), frequency-domain measures (FFT/Welch band powers), and time–frequency representations (DWT/STFT) capture complementary characteristics of ictal dynamics. Nonlinear descriptors—e.g., approximate/spectral/sample entropy and fractal dimension—aim to quantify changes in complexity that often accompany seizures. In practice, systems combine features across domains and feed them to lightweight classifiers. For instance, DWT features with SVMs routinely reach high accuracy on curated benchmarks. Samantaray and Rahulkar [4] employed a bank of separable Gabor wavelets with LDA+SVM and reported 99.10% accuracy (sensitivity 99.02%, specificity 99.18%). Yogarajan et al. [5] used stationary wavelets plus meta-heuristic feature selection before a DNN and reported perfect classification on a benchmark set. Li et al. [6] proposed high-resolution time–frequency “rhythmic encoding” and achieved 98.9% accuracy across 94 patients. These pipelines offer interpretable features, modest computations, and real-time feasibility on standard hardware. Limitations include reliance on manual design choices (wavelet family, bands, thresholds), sensitivity to dataset shifts and artifacts, and challenges with severe class imbalance; consequently, generalization to noisy, continuous clinical EEG can be limited [3].

### 2.2. Machine Learning Methods

Traditional ML augments hand-crafted features with off-the-shelf classifiers such as SVM, Random Forest (RF), KNN, and boosted trees. Alalayah et al. [16] compared multiple classifiers on DWT features (with PCA/t-SNE), reporting 97.96% for RF and 98.98% for an MLP. Wang et al. [17] combined Fourier/STFT descriptors with a swarm-based selector and achieved 96.7% using RF. Among classical methods, RFs often perform strongly on heterogeneous feature sets and require minimal normalization [3]. However, accuracy may degrade on previously unseen patients or locally acquired EEG, underscoring a generalization gap documented by Carvajal-Dossman et al. [3]. Strengths of ML pipelines include interpretability and low latency; their principal weakness is continued dependence on upstream feature engineering and dataset-specific tuning.

### 2.3. Deep Learning Methods

End-to-end deep learning learns hierarchical features from raw or weakly processed EEG, alleviating manual design. One-dimensional CNNs learn temporal filters directly from waveforms; two-dimensional CNNs leverage spectrograms or other time–frequency images; recurrent models (LSTM/GRU) and hybrid CNN-RNNs capture longer temporal dependencies; attention/Transformer modules have also been explored. Zhao et al. [7] combined a ResNet front-end with BiLSTM (ResBiLSTM), reaching 98.9–100% on Bonn and 95.0% on TUSZ. Torkey et al. [8] reported 99.13% with a CNN–LSTM–GRU hybrid and XAI. Other multiscale CNNs and CNN-attention models similarly exceed 90–95% on public datasets [9]. Despite strong benchmark performance, several caveats persist: (i) optimistic cross-validation on pre-segmented data can inflate metrics; (ii) training and inference are compute-intensive; and (iii) cross-patient/site generalization remains challenging [3]. Real-time deployment typically relies on hardware acceleration (e.g., GPUs or TPUs), model compression (e.g., pruning or distillation), or dedicated hardware implementations (e.g., ASICs or FPGAs), with careful attention to end-to-end latency.

### 2.4. Neuromorphic and Spiking Neural Network Methods

Unlike conventional artificial neural networks (ANNs), spiking neural networks (SNNs) operate on discrete spike events and incorporate neuron models that are inspired by biological signal processing principles. In particular, leaky integrate-and-fire (LIF) neurons capture key aspects of neuronal membrane behavior, including temporal integration, leakage, and threshold-based spike generation. Together with event-driven computation, these mechanisms enable sparse temporal processing that is well suited to nonstationary biosignals such as EEG. From a representational perspective, the signal encodings and communication paradigm used in SNN-based systems reflect principles observed in biological sensory pathways. Change-based encoders, such as Delta–Sigma modulation, resemble level-crossing and temporal contrast mechanisms used in sensory transduction, while rate-based encoding represents sustained signal intensity over time. Event-driven spike communication further reflects sparse neural signaling, in which computation is driven by temporally salient activity rather than continuous-valued updates. Accordingly, SNN-based systems are appropriately described as neuromorphic-inspired, exhibiting biomimetic characteristics at the level of neuron dynamics and signal representation rather than constituting detailed biological models.

Recent studies have demonstrated that SNNs can achieve seizure detection performance comparable to ANN-based approaches while offering advantages for low-latency and potentially low-power deployment [12,13]. For example, Zhang et al. [12] reported cross-patient performance competitive with ANN baselines, accompanied by substantial energy reductions on neuromorphic platforms, while Yang et al. [13] achieved approximately 98–99% accuracy on the CHB–MIT dataset [18] by converting trained ANNs to SNNs and deploying them on neuromorphic hardware with reduced power requirements.

Despite these advantages for battery-constrained, always-on monitoring, the practical deployment of SNNs for clinical EEG analysis remains challenging. Open issues include the selection of explicit spike encoding strategies, reliance on surrogate-gradient training or ANN-to-SNN conversion, limited availability of mature neuromorphic hardware, and fragmented software toolchains [12,13]. Moreover, many prior SNN studies are evaluated under simplified or offline conditions, often using long analysis windows, non-causal smoothing, and limited reporting of deployment-critical metrics such as inference latency, spike activity, or computational cost. To address these gaps, the present work introduces a causal hybrid spike encoding scheme and systematically evaluates SNN architectures under real-time streaming conditions, with explicit analysis of spike activity, inference latency, and algorithmic efficiency in a reproducible and clinically relevant framework.

### 2.5. Hybrid and Heuristic Approaches

Hybrid strategies fuse feature families, model types, or optimization heuristics to exploit complementary information. Ren et al. [19] combined AR, wavelet-packet, and entropy features with dimensionality reduction (PCA/mRMR) to reach 99.24% on a benchmark. Sreenivasan et al. [20] proposed a lightweight channel-averaging and masking heuristic that achieved 94.8%. Such systems can reach top-tier accuracy on curated datasets but may introduce complexity and overfitting risks; achieving low-latency, resource-constrained deployment requires careful simplification.

### 2.6. Comparative Analysis and Gaps

Table 1 illustrates recurring trade-offs. Signal processing and ML pipelines remain attractive for their interpretability and efficiency, yet often rely on carefully curated datasets and hand-tuned features; generalization to continuous, artifact-rich clinical EEG is inconsistent [3]. Deep learning achieves state-of-the-art accuracy on many public benchmarks [7,8,9] but demands substantial compute and careful evaluation to avoid optimistic estimates. Neuromorphic SNNs promise substantial energy and latency advantages [12,13], aligning with always-on, battery-constrained monitoring, while still facing training and tooling maturity challenges. Across methods, common gaps include (i) limited patient-wise or site-wise validation; (ii) inconsistent reporting of clinically relevant metrics (e.g., false alarms per hour, detection latency) alongside accuracy; (iii) insufficient treatment of class imbalance in streaming settings; (iv) scarce reporting of runtime and energy; and (v) limited robustness/interpretability analyses.

These observations motivate the present study, which establishes a unified and reproducible framework for benchmarking three complementary paradigms—one-dimensional convolutional neural networks (1D-CNNs), spiking neural networks (SNNs), and convolutional spiking neural networks (ConvSNNs)—under identical preprocessing and evaluation protocols. The proposed pipeline explicitly addresses the trade-offs among accuracy, latency, and energy efficiency that are critical for real-time clinical deployment. In this framework, the baseline 1D-CNN operates in a static, offline manner, processing full-length EEG recordings to detect seizure events. In contrast, the SNN and ConvSNN architectures are designed for real-time operation, processing short, overlapping EEG windows to provide low-latency, event-driven detection suitable for continuous monitoring applications.

## 3. Real-Time Seizure Detection

### 3.1. Dataset, Preprocessing, and Labeling

We employed the publicly available CHB–MIT Scalp EEG Database [18], sampled at 256 Hz. All EEG channels were preprocessed by resolving duplicate labels and removing non-EEG channels such as ECG, EMG, and photic-stimulation markers. Each retained EEG channel was standardized via z-scoring to ensure zero mean and unit variance.

Recordings were segmented into fixed-length windows of 128 samples (corresponding to 0.5 s) with 50% overlap (step size of 64 samples) to support low-latency, streaming-style inference. A window was labeled as seizure if at least 20% of its duration overlapped a clinician-annotated ictal interval, using precise seconds-to-samples mapping. This overlap criterion reduces ambiguity at seizure boundaries and mitigates label noise caused by brief transition periods between ictal and non-ictal states. Similar overlap-based window labeling strategies have been adopted in prior CHB–MIT seizure detection studies (e.g., [23,24]) and in established seizure annotation practices that recommend avoiding boundary contamination [25].

#### 3.1.1. Patient-Wise Grouping and Window Provenance

To enable leakage-free evaluation, each extracted EEG window retains explicit provenance information, including (i) the patient identifier and (ii) the source recording identifier. These identifiers are propagated throughout dataset construction and are used to enforce strictly disjoint train, validation, and test partitions (Section 3.2). As a result, all windows originating from the same recording—including temporally adjacent or overlapping windows—are guaranteed to remain within a single partition. This design eliminates subject-level and temporal leakage during evaluation.

#### 3.1.2. Class Imbalance Handling

Clinical EEG recordings exhibit strong class imbalance, with ictal activity typically accounting for less than 5% of total recording time. To stabilize model optimization, class balancing is applied exclusively to the training partition after data splitting. Specifically, seizure windows are oversampled (with replacement, if necessary), while non-seizure windows are undersampled (without replacement) within the training set only. The validation and test partitions remain unaltered and preserve the natural seizure prevalence, thereby preventing duplicated-window leakage and enabling unbiased performance evaluation.

### 3.2. Train/Validation Separation

Unless otherwise stated, the dataset was partitioned into disjoint subsets using a patient-wise split, with 70% of patients assigned to training and 30% assigned to validation (fixed seed, random_state = 42). All windows from a given patient (and all recordings associated with that patient) are assigned exclusively to a single partition. Formally, if $P_{\mathrm{train}}$ and $P_{\mathrm{val}}$ denote the sets of patient IDs in training and validation, then $P_{\mathrm{train}}\cap P_{\mathrm{val}}=\emptyset$. Consequently, no windows from the same patient, recording, or contiguous EEG segment can appear in both sets, eliminating subject-level and temporal leakage in evaluation. Overlapping windows are retained within each partition to model continuous online processing and reduce missed onsets at window boundaries. However, because the split is enforced at the patient (and recording) level, overlapping or near-duplicate windows cannot cross the train/validation boundary.

Both the Hybrid SNN and ConvSNN models were trained and evaluated using identical patient-wise data partitions and preprocessing pipelines, ensuring that all performance comparisons are strictly matched and methodologically fair. Temperature scaling and decision-threshold sweeps were conducted as post hoc analyses on the validation set solely to characterize operating trade-offs between sensitivity and false-alarm rate. These procedures were not used to retrain, fine-tune, or otherwise optimize model parameters, and reported performance metrics correspond to fixed operating points unless explicitly stated.

### 3.3. Hybrid Spike Encoding: Delta–Sigma + Rate

Each preprocessed EEG window (C×T) (where *C* is the number of EEG channels and *T* is the number of samples in the time window) was transformed into a dual-coded spike tensor by concatenating two complementary event-based encodings, capturing both temporal change and signal intensity.

(i) Delta–Sigma (ΔΣ) Modulation:
  A change-based encoding that emits a spike whenever the instantaneous EEG sample x(t) exceeds a dynamic reference signal r(t) by a fixed step Δ. With Δ=0.3, the encoding rule is
  (1) $$\begin{array}{cc} s(t) & =\left\{\begin{array}{cc} 1, & \mathrm{if}\;x(t)>r(t),\\ 0, & \mathrm{otherwise}, \end{array}\right.\\ r(t+1) & =r(t)+s(t)-\Delta . \end{array}$$
  Here, x(t) denotes the normalized EEG amplitude at time step *t*, r(t) is an adaptive reference tracking recent signal levels, and s(t) is the binary output spike indicating an upward excursion of x(t) beyond r(t). This representation produces sparse, event-driven spike trains that emphasize rapid waveform excursions such as sharp transients, epileptiform spikes, and onset ramps.
(ii) Stochastic Rate Coding: A probabilistic amplitude-based encoding that transforms the normalized EEG signal into a spike train whose firing probability reflects instantaneous signal magnitude [26,27]. Each sample x(t) is mapped to a firing probability:
  (2) $$p\left(t\right)=\sigma \left(x\left(t\right)\right)=\frac{1}{1+e^{-x(t)}},$$
  where σ(·) is the logistic activation. A binary spike b(t) is then generated by sampling from a Bernoulli process,
  (3) b(t)∼Bernoulli(p(t)),
  so that b(t)=1 with probability p(t) and b(t)=0 otherwise. This stochastic process produces a sequence of independent binary events whose average firing rate approximates the input amplitude. Consequently, sustained or high-amplitude EEG activity yields denser spike trains, preserving signal energy and rhythmic oscillations while maintaining compatibility with event-driven neuromorphic computation.

**Figure 1:**
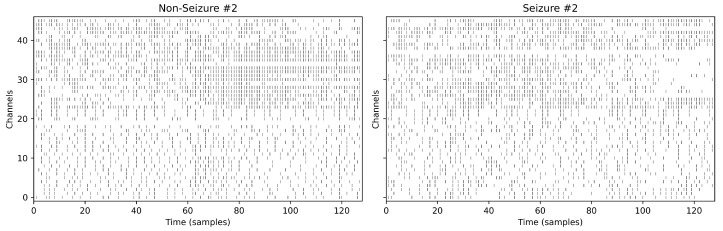
Spike-encoded representations of EEG windows (128 samples, 0.5 s). Spikes were generated using the hybrid Delta–Sigma and stochastic rate encoding method described in Section 3.3. Channels indexed 0–22 correspond to Delta–Sigma encoded spikes, while channels indexed 23–45 correspond to rate-encoded spikes derived from the same EEG inputs.

For each EEG channel, both s(t) and b(t) are generated and concatenated along the channel dimension, producing a hybrid spike tensor of shape (2C×T). Figure 1 illustrates representative 2C×T EEG windows for non-seizure and seizure recordings. This dual representation jointly encodes differential (change-based) and integral (rate-based) features of the EEG, enabling downstream spiking neural networks to integrate fast transient events with slower rhythmic patterns. Empirically, ablation studies confirmed that either encoding alone underperformed the hybrid scheme, underscoring the complementary nature of the two representations.

### 3.4. Models

#### 3.4.1. Training Methodology

All spiking neural network models in this study are trained using surrogate-gradient backpropagation, which is the standard training approach supported by modern SNN frameworks such as SNNtorch [28]. While learning rules based on spike-timing-dependent plasticity (STDP) and Hebbian mechanisms have been explored in supervised or reward-modulated settings, their use for training deep spiking architectures with convolutional and attention-based components and end-to-end task-specific loss functions is not yet a standardized practice. Surrogate-gradient learning therefore provides a practical and stable solution for supervised optimization while preserving spike-based inference dynamics.

#### 3.4.2. One-Dimensional-CNN Baseline

A one-dimensional convolutional neural network (1D-CNN) was implemented as a baseline to provide a reference point for seizure detection performance using conventional dense neural computation. Unlike the proposed spiking models, the CNN operates in an offline setting and is trained on full-length EEG recordings rather than short, causal windows. The CNN baseline is included as a contextual reference to illustrate achievable performance under offline analysis conditions with access to extended temporal context. Its use of dense multiply–accumulate operations and continuous-valued activations enables strong classification accuracy, but it operates under different input granularity and latency assumptions than the proposed spiking models. Accordingly, the CNN does not share the same operational constraints as the Hybrid SNN and ConvSNN, which process short 0.5 s windows in a fully causal, streaming manner. The CNN results are therefore reported to contextualize offline performance rather than to provide a directly matched real-time comparison.

The 1D-CNN architecture consists of three temporal convolutional blocks followed by global average pooling and two fully connected layers. Each convolutional block extracts hierarchical temporal features from long EEG sequences using dense matrix operations, as summarized in Table 2.

The model is trained using binary cross-entropy loss and the Adam optimizer, achieving rapid convergence and strong classification accuracy on balanced data. However, its dense operations and continuous activations result in a high computational cost and significant memory footprint. Each inference requires processing thousands of time samples across multiple convolutional layers, which is not compatible with energy-constrained or implantable systems that must operate continuously.

In contrast, the proposed spiking models—both the feed-forward SNN and the convolutional SNN (ConvSNN)—operate in an online, causal manner, processing 0.5 s EEG windows with 50% overlap. By converting analog EEG signals into sparse spike events and updating internal states sequentially, these models support low-latency inference under streaming conditions. Accordingly, while the 1D-CNN serves as an offline performance reference, the SNN and ConvSNN are designed for real-time, event-driven operation.

#### 3.4.3. Hybrid SNN (Feed-Forward, Dual-Encoded Input)

The Hybrid SNN processes the dual-coded spike tensor (2C×T) that combines both ΔΣ (change-based) and rate (amplitude-based) spike trains for each EEG channel. At each time step, the network applies a linear projection followed by layer normalization and an LIF activation equipped with surrogate-gradient learning. Dropout regularization and a final linear classification layer are subsequently applied. Temporal information is integrated by accumulating and averaging per-step logits across all *T* time steps to generate final class probabilities, which are optimized using cross-entropy loss.

**Figure 2:**
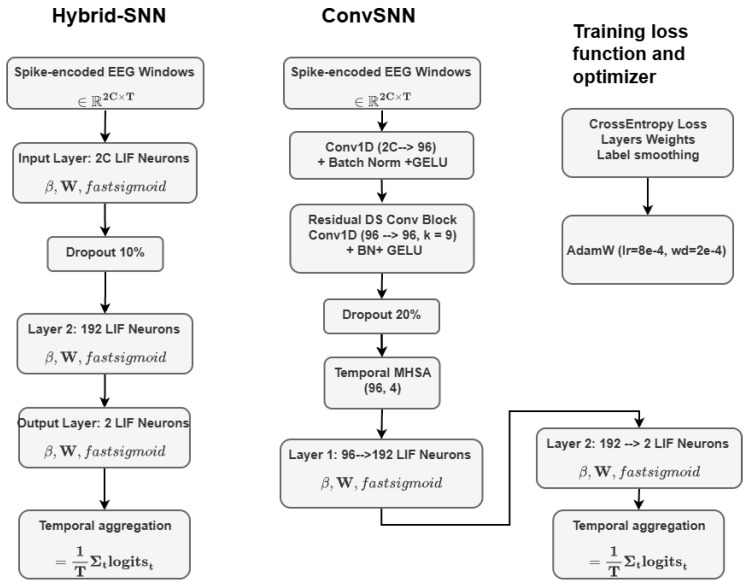
Block-diagram comparison of the two proposed spiking architectures. Left: HybridSNN—A fully connected spiking network operating on dual-encoded spike representations (Delta–Sigma and stochastic rate). The model performs temporal evidence aggregation using time-averaged logits and is trained via weighted cross-entropy with label smoothing and one-cycle learning-rate scheduling. Right: ConvSNN-ResAttn—A convolutional spiking architecture with (i) an optional latency-coded spike stream, (ii) a residual depthwise-separable convolutional front-end, (iii) temporal multi-head self-attention, and (iv) a spiking classification head. The pipeline includes calibration (temperature scaling) and post hoc threshold sweeping on seizure probability for optimal F1/accuracy trade-off. Both models operate on spike-encoded EEG windows of 0.5 s (128 samples) and follow the same training pipeline of gradient clipping and early stopping.

The hidden layer (Layer 2) dimension was set to $n_{\mathrm{hidden}}=192$ with a dropout probability of 0.1, and the LIF decay parameter β was initialized at 0.97 and treated as a learnable parameter during training. Optimization employed the AdamW algorithm with a OneCycle learning-rate policy. This configuration preserves the architectural simplicity of a shallow SNN while fully exploiting the complementary temporal and amplitude information embedded in the hybrid spike representation. Figure 2 (left) shows the block diagram of the Hybrid-SNN architecture.

#### 3.4.4. ConvSNN (Residual Temporal Convolutions + MHSA + Spiking Head)

To capture longer-term temporal patterns and inter-channel structure, the ConvSNN first applies a Conv1D stem (width 64–96, k=3), followed by two residual depthwise-separable temporal blocks (k=9, strides (1,2)). A lightweight temporal multi-head self-attention (MHSA) module with four heads aggregates global context. Finally, a spiking head (FC–LIF–dropout–FC–LIF) integrates information over time and outputs time-averaged logits for cross-entropy loss. The LIF decay parameter β is parameterized as β=exp(−Δt/τ), with τ≈20 ms, and is learned during training. Optimization uses AdamW with a OneCycleLR schedule, gradient clipping (∥g∥≤1.0), and the same label smoothing and class weighting strategy as described in Section 3.4.3. Figure 2 (right) illustrates the ConvSNN architecture.

Table 3 outlines the configurations and parameter counts of the proposed Hybrid SNN and ConvSNN models. Both architectures process 46-dimensional dual-coded spike inputs (23 EEG channels with Delta–Sigma and rate encoding). The Hybrid SNN adopts a compact feed-forward structure with a single hidden layer of 192 neurons, leaky integrate-and-fire (LIF) dynamics, and fewer than 10k parameters, enabling efficient real-time inference. The ConvSNN extends this design with a convolutional stem, two residual depthwise-separable convolutional blocks, and a temporal multi-head self-attention module for enhanced temporal representation. Despite its higher parameter count, the ConvSNN remains lightweight and suitable for online, low-latency EEG seizure detection.

## 4. Results

### 4.1. Performance Overview

Table 4 summarizes the operating-point performance of the evaluated architectures, including an offline 1D–CNN baseline, a feed-forward SNN, and the proposed ConvSNN. Reported metrics correspond to the validation partition, with operating thresholds selected as described in Section 3.2. The 1D–CNN achieved the highest accuracy (99.3%) and F1-score (0.985) when trained on full-length EEG recordings. This performance benefits from dense convolutional processing and access to extended temporal context across entire recordings. As such, the CNN serves as an offline upper-bound reference rather than a directly comparable real-time baseline, as it does not support causal inference or low-latency deployment. In contrast, both the SNN and ConvSNN are trained and evaluated using short, causal 0.5 s EEG windows processed in a streaming manner. The hybrid spike representation, combining Delta–Sigma and rate encoding, allows both models to capture clinically relevant temporal structure despite sparse input activity. The SNN achieves 91.8% accuracy with an F1-score of 0.834, while the ConvSNN—which incorporates residual depthwise-separable convolutions and temporal multi-head self-attention prior to its spiking classification head—achieves 94.7% accuracy and an F1-score of 0.8934. This corresponds to an approximate 7.1% relative improvement in F1-score over the simpler SNN, indicating that additional temporal feature modeling improves detection performance under the same causal and windowed constraints.

### 4.2. Confusion Matrix Analysis

**Figure 3:**
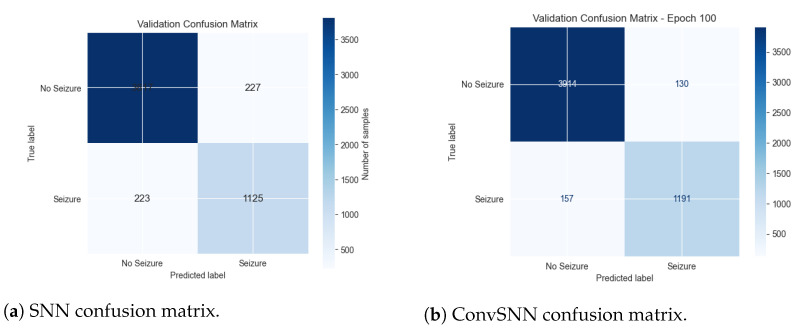
Validation confusion matrices for (**a**) the SNN and (**b**) the ConvSNN. Both models show strong discrimination between seizure and non-seizure windows, with the ConvSNN demonstrating higher sensitivity and reduced false positives.

Figure 3 illustrates the confusion matrices for the SNN and ConvSNN models. Both models demonstrate strong discrimination between seizure and non-seizure windows, though the ConvSNN yields notably fewer false negatives (missed seizures), an important property for clinical reliability. Specifically, the ConvSNN achieved 1191 true positives compared to 1125 in the SNN, while simultaneously reducing false positives (130 vs. 227), resulting in both higher sensitivity (recall) and precision.

### 4.3. Discussion of Real-Time Viability

Although the 1D–CNN achieves the highest accuracy among the evaluated models, its dependence on dense floating-point computation and long temporal context makes it unsuitable for real-time deployment or resource-constrained edge devices. In contrast, both the Hybrid SNN and the ConvSNN operate in an event-driven manner, processing each 0.5 s EEG window independently without relying on future context. This strictly causal design aligns their algorithmic latency with clinical requirements for continuous bedside monitoring. Both models generate decisions within a few milliseconds per 0.5 s window, enabling real-time inference on standard hardware and positioning the approach for future integration into neuromorphic accelerators. The ConvSNN offers a compelling balance between accuracy and efficiency, demonstrating the value of combining spiking dynamics with lightweight convolutional processing and temporal attention in event-driven EEG analysis.

#### 4.3.1. Measured Inference Latency

To quantify real-time performance, we measured the inference time on a standard desktop CPU (Intel Core i7, 3.8 GHz, no GPU). Processing a batch of 64 windows required 78.345 ms for the Hybrid SNN and 73.991 ms for the ConvSNN. Since each batch contains 64 independent 0.5 s windows, the per-window latency is

$$t_{\mathrm{HybridSNN}}=\frac{78.345\;\mathrm{ms}}{64}=1.224\;\mathrm{ms},\;t_{\mathrm{ConvSNN}}=\frac{73.991\;\mathrm{ms}}{64}=1.156\;\mathrm{ms}.$$

Both latencies are more than two orders of magnitude faster than the 0.5 s window duration. Thus, both models operate far beyond real-time on commodity CPU hardware, requiring no GPU acceleration and comfortably supporting embedded or hospital-deployed monitoring systems.

#### 4.3.2. Model Comparison

Performance comparisons focus on metrics relevant to real-time inference, including window-level accuracy, F1-score, and inference latency per window. In addition, computational characteristics such as parameter count and event-driven sparsity are reported to highlight differences between dense and spiking architectures. These criteria reflect the primary goal of assessing suitability for real-time event-driven EEG inference, rather than offline batch classification.

The ConvSNN exhibits the best trade-off between latency, efficiency, and detection performance. Its combination of convolutional processing, temporal self-attention, and spiking dynamics improves sensitivity while maintaining an extremely low computational cost. This highlights the practical advantage of hybrid event-driven architectures for low-latency EEG analytics.

Representative end-of-training results (window level) are summarized as

- HybridSNN: Accuracy 0.913±0.004, F1-score 0.8333; confusion matrix demonstrates balanced sensitivity and specificity.
- ConvSNN: Accuracy 0.9470±0.009, F1-score 0.8934; with post hoc selected thresholds τ≈0.66–0.76.

**Figure 4:**
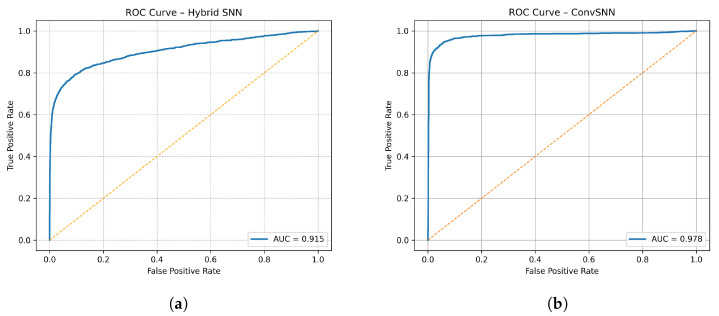
ROC curves are reported at the window level; false-alarm rates and detection latency are evaluated separately using event-level alarm logic. (**a**) ROC curve for the Hybrid SNN model computed using window-level seizure probabilities. The area under the curve (AUC = 0.915) indicates strong discrimination between seizure and non-seizure EEG windows across decision thresholds. (**b**) ROC curve for the ConvSNN model computed using window-level seizure probabilities. The higher area under the curve (AUC = 0.978) reflects improved discriminative performance resulting from convolutional and temporal-attention processing.

Figure 4 presents the receiver operating characteristic (ROC) curves for the Hybrid SNN and ConvSNN models, computed by sweeping the decision threshold over window-level seizure probabilities. The Hybrid SNN achieves an ROC-AUC of 0.915, indicating strong discriminative capability between seizure and non-seizure EEG windows. The ConvSNN further improves discrimination, achieving an ROC-AUC of 0.978, reflecting the benefit of additional convolutional and temporal-attention processing while retaining low-latency, event-driven operation. These results demonstrate that both architectures provide reliable threshold-independent separation of seizure activity, enabling the selection of operating points that balance sensitivity and false-alarm rates under streaming alarm logic. While ROC analysis evaluates window-level discrimination, clinical usability is assessed separately through event-level false-alarm rates and detection latency after temporal aggregation.

#### 4.3.3. Clinical Relevance and False-Alarm Analysis Under Streaming Evaluation

Because the Hybrid SNN and ConvSNN produce one decision per 0.5 s EEG window, the proposed system operates in a streaming mode, updating seizure likelihood at an effective rate of 2 Hz. In clinical practice, however, seizure alarms are not triggered by isolated window-level detections. Instead, sustained ictal activity over multiple seconds is typically required to suppress transient artifacts and reduce false positives. Accordingly, practical seizure detection systems apply temporal aggregation or persistence rules to window-level outputs before raising an alarm. In this work, false-alarm performance is evaluated using continuous EEG recordings from the CHB–MIT dataset, processed sequentially in time on a per-patient basis. All available EEG recordings for each patient are replayed in chronological order, without window shuffling, class balancing, or resampling. Importantly, the evaluation stream reflects the natural seizure prevalence present in the original recordings and does not use the balanced training distribution. Window-level predictions are aggregated using a conservative temporal persistence rule to emulate realistic seizure-alarm behavior. With 0.5 s windows and 50% overlap, annotated seizures in CHB–MIT—whose minimum duration is approximately 10 s—span at least 39 consecutive windows. A seizure alarm is therefore declared when at least 10 consecutive windows are classified as seizure, corresponding to a minimum persistence of approximately 2.5 s. This rule enables early detection while effectively suppressing isolated false positives caused by noise or brief artifacts. False alarms are defined as alarm events that do not overlap any clinician-annotated ictal interval. The false-alarm rate is computed by counting the total number of such events over the full duration of continuous EEG recordings and normalizing by the total recording time, yielding false alarms per hour (FA/h) and per day (FA/day). Under this streaming—event-level evaluation—the Hybrid SNN achieves an average false-alarm rate of 0.034 FA/h, corresponding to 0.82 FA/day, while the ConvSNN achieves 0.026 FA/h, corresponding to 0.62 FA/day. These values fall within commonly cited clinical acceptability thresholds (approximately one false alarm per day), indicating that the proposed spiking models maintain practical usability when deployed for continuous EEG monitoring.

#### 4.3.4. Operational Interpretation of Window-Level Results

While classification performance is reported at the window level for analytical clarity, clinical seizure detection systems do not trigger alarms based on isolated window-level decisions. Instead, consecutive positive detections are aggregated over time to ensure persistence of ictal activity and to suppress transient false positives. Consistent with the streaming evaluation described in Section 4.3.3, operational seizure alarms in this work are derived using temporal aggregation of window-level outputs rather than single-window decisions. As a result, window-level false positives should be interpreted as intermediate indicators of model behavior, whereas clinically relevant false-alarm rates are quantified using event-level metrics such as false alarms per hour and per day computed over continuous EEG recordings.

#### 4.3.5. Ablation: Effect of Hybrid Encoding

Using only Delta–Sigma encoding emphasizes abrupt waveform transitions but underrepresents sustained ictal activity, while rate-only encoding captures amplitude dynamics but tends to blur sharp temporal changes. We therefore began our investigation by designing a compact feed-forward SNN intended for low-latency, low-complexity seizure detection and evaluated its performance using single-stream spike encodings. As shown in Table 5, neither Delta–Sigma nor rate encoding alone yielded satisfactory detection performance under the strict patient-wise, causal evaluation adopted in this study. Validation F1-scores remained below 0.70 for the SNN, indicating limited separability when relying on a single temporal representation. Motivated by these observations, we introduced a hybrid encoding strategy that concatenates Delta–Sigma and rate representations within the same lightweight SNN architecture (HybridSNN). This design substantially improves detection performance by exposing complementary temporal information to spiking neurons: change-based spikes highlight rapid waveform transitions, while rate-based spikes capture sustained amplitude and rhythmic patterns characteristic of seizures. Importantly, this performance gain is achieved without increasing window length or relying on non-causal context, preserving suitability for real-time operation. Building on the effectiveness of hybrid encoding, we subsequently extended the architecture with lightweight convolutional and temporal-attention components (ConvSNN), achieving further improvements while maintaining a compact model footprint. Overall, these ablation results provide direct empirical evidence that hybrid spike encoding is a key factor enabling accurate real-time seizure detection in resource-constrained wearable and clinical monitoring scenarios.

#### 4.3.6. Training Dynamics and Generalization

Both spiking models exhibited stable convergence when trained with surrogate gradients and label smoothing. The OneCycleLR scheduler accelerated optimization and mitigated overfitting plateaus. Mild seizure up-weighting (1.2×) improved recall by approximately 1–2% with negligible impact on precision. The ConvSNN’s inclusion of residual depthwise-separable convolutions and temporal multi-head self-attention (MHSA) consistently yielded 2–4% relative gains in F1-score compared to the simpler Hybrid SNN.

#### 4.3.7. Computational Complexity and Spike-Activity Analysis

Energy efficiency is a key motivation for spiking neural networks in continuous EEG monitoring, particularly for edge and wearable systems. As the present study does not include deployment on neuromorphic hardware, computational efficiency is assessed at the algorithmic level using spike-activity statistics and architectural complexity, which are commonly adopted proxies in SNN research. Spike activity is analysed at the input encoding level. For each 0.5 s EEG window (128 samples at 256 Hz), signals from C=8 EEG channels are converted into binary spike trains using Delta–Sigma encoding, rate encoding, or their combination. Spike density is defined as the fraction of channel–timestep bins within a window that contain a spike event, averaged across all windows:

$$\rho =\frac{\#\;\mathrm{spike}\;\mathrm{events}}{B\times C\times T},$$

where *B* is the number of windows, *C* the number of channels, and *T* the number of timesteps per window. Importantly, this is a per-channel, per-timestep occupancy probability, not an aggregate firing rate across channels or layers. Table 6 reports average input spike density computed over 5392 validation windows using this definition. The resulting densities (ρ≈0.054–0.059) indicate that each EEG channel emits spikes at approximately 5–6% of timesteps. When converted to an equivalent firing rate, this corresponds to roughly 14–15 spikes per second per channel, given the 256 Hz sampling rate. These values reflect sparse, event-driven input activity at the channel level and are consistent with spike rates commonly reported in temporal SNN signal processing applications. The substantially higher values reported in earlier preliminary analyses were attributable to an alternative normalization that did not account for the channel dimension; the present definition reflects the correct per-channel spike occupancy.

## 5. Discussion

### 5.1. Rationale for Hybrid Signal-to-Spike Conversion

Epileptic seizures are characterized by both rapid, transient discharges and sustained rhythmic oscillations. The Delta–Sigma encoder is particularly effective for the former, emitting sparse spikes at each level crossing to highlight sudden changes while suppressing redundancy. Rate coding, in contrast, represents amplitude through probabilistic firing, effectively encoding tonic or rhythmic activity. By concatenating both encodings, the hybrid representation provides complementary evidence streams that the spiking network can integrate over time. Empirically, this dual representation improved discriminative performance, demonstrating higher F1-scores than either encoding alone.

### 5.2. Architectural Rationale: Hybrid SNN vs. ConvSNN

The Hybrid SNN serves as a minimal spike-based architecture that validates the effectiveness of hybrid spike encoding under causal, low-latency constraints. It relies on simple linear transformations and leaky integrate-and-fire (LIF) dynamics to perform temporal evidence integration. While this design achieves competitive performance with low computational overhead, its limited depth restricts the ability to model structured rhythmic patterns and inter-channel relationships in EEG signals.

The ConvSNN extends this baseline by introducing residual depthwise-separable temporal convolutions to capture local temporal structure and by incorporating multi-head self-attention (MHSA) to model longer-range temporal dependencies and emphasize informative signal segments. These additions increase representational capacity while maintaining efficient inference, resulting in improved seizure discrimination under real-time streaming conditions.

### 5.3. Accuracy–Efficiency Trade-Offs

The Hybrid SNN and ConvSNN operate on sparse event streams using leaky integrate-and-fire (LIF) dynamics and process short, causal EEG windows under streaming constraints. The Delta–Sigma encoding emphasizes signal changes and reduces spike activity at the input level, leading to lower activation density.

The Hybrid SNN adopts a compact architecture with low computational demand, achieving reasonable detection accuracy while maintaining very low inference latency. This design makes it well aligned with real-time, low-power-oriented seizure detection scenarios under streaming constraints. The ConvSNN extends this baseline by introducing separable convolutions and temporal attention, increasing representational capacity and improving detection performance at the cost of higher architectural complexity. Together, these results illustrate a clear trade-off between accuracy and model complexity within spiking architectures.

### 5.4. Limitations and Future Work

The present study focuses on causal, patient-wise seizure detection using short (0.5 s) EEG windows processed in a streaming manner. While a strict patient-wise split is employed to prevent subject-level and temporal leakage, the evaluation is conducted under a within-patient monitoring scenario, reflecting continuous bedside or wearable deployment rather than cross-patient generalization. Assessing robustness under leave-one-patient-out or cross-institutional settings remains an important direction for future work. To stabilize training under severe class imbalance, the training set is balanced using resampling, whereas all evaluation metrics—particularly false alarms per hour/day—are computed on continuous EEG recordings with natural seizure prevalence. Although this separation avoids optimistic bias, prospective validation on longer, uninterrupted recordings with clinician-defined alarm criteria would further strengthen clinical relevance. Finally, computational efficiency is assessed using algorithmic proxies (spike activity statistics and measured inference latency) rather than direct hardware power measurements. While these results indicate suitability for real-time deployment, energy profiling on neuromorphic or embedded hardware platforms (e.g., FPGA or ASIC implementations) is left to future work. Additional extensions include adaptive multiscale encoding, patient-adaptive calibration, and hardware-aware optimization of convolutional and attention components.

## 6. Conclusions

This study presented a hybrid spike encoding framework that combines Delta–Sigma and stochastic rate coding with two spiking neural architectures—the Hybrid SNN and the ConvSNN—for EEG-based epileptic seizure detection. Both models operate on short, causal 0.5 s EEG windows and support real-time, streaming inference through event-driven signal processing. When evaluated on the CHB–MIT dataset using a strict patient-wise protocol, the Hybrid SNN achieved an accuracy of 0.917 and an F1-score of 0.833, while the ConvSNN achieved an accuracy of 0.948 and an F1-score of 0.893. These results demonstrate that explicit hybrid spike encoding, together with structured temporal processing, enables robust seizure detection under low-latency constraints.

From a biomimetic engineering perspective, the proposed approach draws on abstract principles observed in biological sensory information processing—namely event-driven signal representation, threshold-based temporal integration, and causal accumulation of evidence over time. These principles are applied at the level of signal encoding and computational dynamics, rather than through detailed biological modeling, to inform an engineering-oriented design for real-time EEG analysis. The work therefore emphasizes algorithmic robustness, interpretability, and computational efficiency, while avoiding claims of biological fidelity.

Taken as a whole, the findings highlight the effectiveness of hybrid spike-based representations and lightweight temporal modeling for reliable, real-time seizure detection in continuous monitoring scenarios, and support the use of principle-level biomimetic abstractions as a practical design strategy for time-critical biomedical signal processing systems.

## Figures and Tables

**Table 1:** Representative recent studies in automated EEG seizure detection. Reported metrics are as stated by the original authors and are not directly comparable due to differences in datasets, evaluation protocols, and operational assumptions (offline vs. real-time).

Reference	Method	Dataset(s)	Key Reported Metrics
Samantaray [4]	Gabor wavelets + LDA + SVM	Benchmark (reported)	Acc 99.10%; Sens 99.02%; Spec 99.18%
Yogarajan et al. [5]	Stationary wavelet + meta-heuristic selection + DNN	Benchmark (reported)	Acc 100%
Li et al. [6]	High-resolution time–frequency rhythmic encoding	94 patients (authors’ dataset)	Acc 98.9% (93/94 seizures detected)
Alalayah et al. [16]	DWT + PCA/t-SNE + RF/MLP	Public benchmarks	RF 97.96%; MLP 98.98%
Wang et al. [17]	1D-CNN + meta-heuristic feature selection	Public benchmarks	Acc 96.7%
Zhao et al. [7]	ResNet + BiLSTM (ResBiLSTM)	Bonn, TUSZ	Bonn 98.9–100%; TUSZ 95.0%
Torkey et al. [8]	CNN–LSTM–GRU with explainability	Balanced test sets	Acc 99.13%
Jia et al. [9]	Multiscale CNN variants	Bonn, CHB–MIT	∼99% (Bonn); >95% (CHB–MIT)
Zhang et al. [12]	Event-driven convolutional/ recurrent SNN (EESNN)	CHB–MIT, others	ANN-comparable accuracy with orders-of-magnitude energy reduction
Yang et al. [13]	ANN→SNN conversion on neuromorphic hardware	CHB–MIT (reported)	∼98–99% accuracy with substantial energy savings
Sreenivasan et al. [20]	Channel-averaged mask heuristic	Public benchmarks	Acc 94.8%
Carvajal-Dossman et al. [3]	Systematic ML/DL re-evaluation study	Multiple datasets	Significant performance drops on local EEG
Berrich et al. [21]	CNN–SVM/DNN–SVM with PCA	Benchmark (reported)	High accuracies (reported)
Zhang et al. [22]	Attention-fusion SNN (DAFF-SNN)	Benchmark (reported)	High accuracies (reported)

**Table 2:** Architecture of the 1D-CNN baseline model.

Layer (Type)	Output Shape	Parameters
Conv1D (32 filters, kernel = 3)	(None, 2556, 32)	192
MaxPooling1D (pool = 2)	(None, 1278, 32)	0
Conv1D (64 filters, kernel = 5)	(None, 1274, 64)	10,304
MaxPooling1D (pool = 2)	(None, 637, 64)	0
Conv1D (128 filters, kernel = 3)	(None, 635, 128)	24,704
GlobalAveragePooling1D	(None, 128)	0
Dense (64 units, ReLU)	(None, 64)	8256
Dense (1 unit, Sigmoid)	(None, 1)	65
Total parameters		43,521

**Table 3:** Architectures of the Hybrid SNN and ConvSNN models. Both operate on 46-dimensional dual-coded spike inputs (23 EEG channels × 2 encodings) over 0.5 s windows in real-time.

Layer (Type)	Output Shape	Parameters
Hybrid SNN		
Linear (46 → 192) + LayerNorm + LIF + Dropout(0.20)	(None, 192)	9792
Linear (192 → 2) + LIF (output)	(None, 2)	196
Total parameters (Hybrid SNN)		9988
ConvSNN (ResDS + MHSA)		
Conv1D (46 → 96, k = 3) + BN + GELU (Stem)	(None, 96, *T*)	13,440
Residual DS-Conv Block 1 (96 → 96, k = 9) + BN + Dropout	(None, 96, *T*)	10,464
Temporal Multi-Head Self-Attention (96, 4 heads) + LN	(None, 96, *T*)	37,440
FC (96 → 192) + LN + LIF + Dropout(0.20)	(None, 192)	19,200
FC (192 → 2) + LIF (output)	(None, 2)	388
Total parameters (ConvSNN)		80,932

**Table 4:** Comparison of model performance on the CHB–MIT validation dataset. The 1D–CNN operates offline with extended temporal context, whereas the SNN and ConvSNN operate in real-time on causal 0.5 s dual-coded spike windows. Reported metrics correspond to validation-set operating points; decision thresholds were selected post hoc to examine operating trade-offs. Loss values are omitted due to non-comparable training dynamics; error rate (1−Accuracy) is reported instead.

Model	Accuracy	Error Rate	Precision	Recall	F1-Score
1D–CNN (offline)	0.9932	0.0068	0.9800	0.9980	0.9850
SNN	0.9177	0.0823	0.8321	0.8345	0.8333
ConvSNN	0.9470	0.0530	0.8975	0.8895	0.8934

**Table 5:** Ablation study of spike encoding strategies under patient-wise, causal evaluation. Reported values correspond to validation performance.

Model	Encoding	Accuracy	F1-Score
SNN	Delta–Sigma only	0.748	0.671
SNN	Rate only	0.780	0.694
HybridSNN	Delta–Sigma + Rate	0.918	0.834
ConvSNN	Delta–Sigma only	0.823	0.701
ConvSNN	Rate only	0.866	0.747
ConvSNN	Delta–Sigma + Rate	0.947	0.893

**Table 6:** Input spike activity measured over all validation windows. Spike density is defined as the fraction of timesteps within a 0.5 s window at which a given EEG channel emits a spike. Equivalent firing rates assume a sampling rate of 256 Hz.

Encoding Stream	Spike Density ρ	Firing Rate (Spikes/s/Channel)
Delta–Sigma (change-based)	0.0589	15.1
Rate-coded	0.0538	13.8
Hybrid (Delta–Sigma + rate)	0.0564	14.4

## Data Availability

Codes are avaiable at [29].
